# Prognosis elements in surgical treatment of complicated umbilical hernia in patients with liver cirrhosis

**Published:** 2013-09-25

**Authors:** P Banu, F Popa, VD Constantin, C Bălălău, M Nistor

**Affiliations:** * General Surgery Department, “Sf. Pantelimon" Emergency Hospital, Bucharest; ** "Carol Davila" University of Medicine and Pharmacy, Bucharest; General Surgery Department, “Sf. Pantelimon" Emergency Hospital, Bucharest

**Keywords:** liver cirrhosis, umbilical hernia, complications

## Abstract

**Introduction: **The surgical treatment of umbilical hernia in cirrhosis patients raises special management challenges. The attitude upon the repair of these hernias varies from expectancy or elective treatment in early stages of the disease to the surgical treatment only if complications occur.

**Material and Method:** We have assessed 22 consecutive cases of cirrhosis patients treated for complicated umbilical hernia in the Surgical Department of “Sf. Pantelimon" Emergency Hospital in Bucharest between January 2008 and December 2012. The patients’ stratification was done in stages of liver disease based upon Child-Pugh classification. Complications that required emergency repair were the following: strangulation, incarceration and hernia rupture. The postoperative complications were ordered in five grades of severity based upon Clavien classification.

**Results: **The severity of the complications was higher in advanced stages of liver cirrhosis, Child B and C. There were 5 deaths representing 22,7%, four of them in patients with Child C disease stage.

**Conclusion:** The incidence of morbidity and mortality after umbilical hernia repair in emergencies increases in advanced stages of liver cirrhosis. It is advisable to prevent complications occurrence and perform surgical repair of umbilical hernia in elective condition.

## Introduction

Liver cirrhosis is a disease whose incidence is estimated at a rate between 5 and 20 in the general population of the East European countries. Mostly affecting people of working age, it is a serious public health problem [**1**]. 

 The main etiological factors of the disease are hepatitis virus infection and alcohol abuse [**2**]. The disease is progressive in nature and is characterized by hepatocyte necrosis followed by fibrosis repair process so the hepatic architecture is deeply affected. The result is a disruption of the circulatory flow and an isolation of the hepatocytes in the regenerative nodules, changes that reach to compromise liver function [**3**]. 

Advanced stages of the disease are characterized by the presence of signs of portal hypertension and hepatocyte failure.

 Deficiency in protein synthesis, coagulation disorders, immunological disturbances, in addition to cardiac dysfunction, respiratory and lung disease associated with late stages make the patient with liver cirrhosis a "difficult field" for surgery [**4**]. 

 The incidence of parietal defects and related morbidity are high in patients with liver cirrhosis, with a significant increase in disease progression. If the umbilical hernia patients without ascites incidence is similar to that in the general population, the occurrence of ascites, especially the tense ascites will be associated with the presence of umbilical hernia in 30-40% of these patients [**5**]. 

 The increased incidence of umbilical hernia is correlated with the patient’s numbers of ascites episodes developed over time, reaching an incidence of 70% in the third episode of ascites [**6**]. 

 Hernia in patients with liver cirrhosis is due to a combination of factors such as increased abdominal pressure by the presence of tense ascites and fascial and muscular structures weakness because of nutritional status [**5**]. 

 Complications related to these hernias are severe. Vascular disorders in the skin region overlying the hernial sac can go up until an ulceration that can cause life-threatening peritonitis appears. Strangulation is another complication of these hernias and it is especially due to paracentesis [**7**]. 

 The indications of elective hernia surgery in patients with liver cirrhosis are for the improvement of the quality of life, regardless of disease stage, with results comparable to patients without cirrhosis [**8**]. 

 The occurrence of a complication requires a surgical solution in the emergency conditions and the possibilities to correct the general disorders are limited mainly by the time factor. In this paper, we tried to evaluate the results in terms of mortality and morbidity in patients with liver cirrhosis in emergency surgery for the umbilical hernia complicated to determine the prognosis related to the evolutionary stage of liver cirrhosis.

## Material and Method

**Fig. 1:**
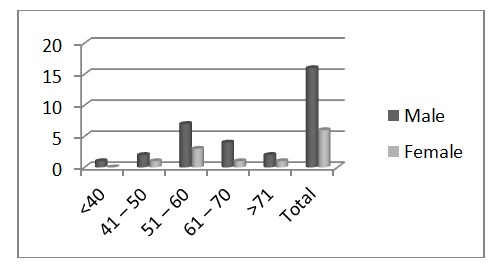
Male / female ratio in the study group

22 consecutive cases of patients with liver cirrhosis treated in the Surgery Clinic of "St. Pantelimon" Emergency Hospital Bucharest between January 2008 and December 2012 for complicated umbilical hernia were reviewed. The average age of the study group was 56.8 years, with a male / female ratio of 2.66 /1,16 men and 6 women. See **Fig. 1**.

Distribution according to age is shown in **Table 1**. The age range was between 38 and 74 years.

**Table 1:** Distribution according to age

Age	Male	Female
<40	1	0
41 – 50	2	1
51 – 60	7	3
61 – 70	4	1
>71	2	1
Total	16	6

The etiology of cirrhosis was identified in these patients as hepatitis C infection in 6 cases, 4 cases of hepatitis B, alcohol abuse in 10 cases. In 2 cases, the etiology of the liver disease could not be documented and it was labeled as cryptogenic. 

**Fig. 2:**
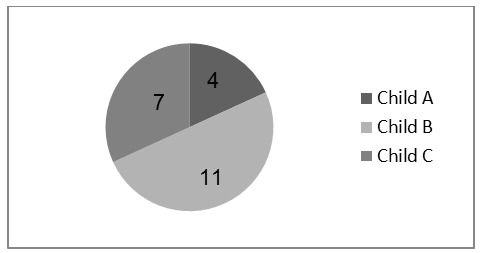
Child-Pugh-Turcotte classification

Regarding the evolutionary stage of liver cirrhosis, patients’ stratification was based on classification Child-Pugh-Turcotte. Under such assignment, 4 patients ranged in group A (18.2%), 11 in group B (50%) and 7 in group C (31.8%). See **Fig. 2**.

Complications of hernia that required surgery were: the strangulation in 11 cases (8 men and 3 women), incarceration - 6 cases (5 men and one woman) and peritoneo-cutaneous fistula with skin ulceration of the hernia - 5 cases (3 men and 2 women).

**Fig. 3:**
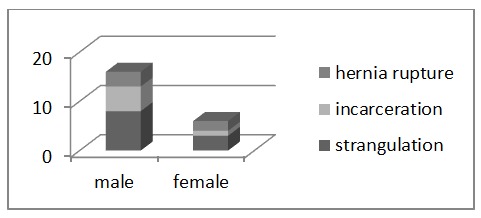
Partial omentectomia

In cases of strangulation, the saccular content was small intestinal loops in 5 cases, two of them presenting advanced parietal lesions that required bowel resection with transit restoring by mechanical anastomosis. The omentum strangulation was found in 6 cases. All these latter cases underwent partial omentectomia. See **Fig. 3**.

Surgical procedures were practiced under local anesthesia (17 cases), or spinal anesthesia (5 cases). Cases were managed by using a two-layer repair technique – 7 cases and one layer technique – 15 cases of interrupted sutures using polypropylene 1.

Patients were followed for 30 days postoperatively.

Complications recorded in these patients were divided into five categories according to their severity after Clavien-Dindo classification [**9**].

Grade 1 complications were those that required symptomatic treatment (analgesics, antispasmodic, and antiemetic) or simple maneuvers, performed without anesthesia to the bedside - evacuation of seroma, hematoma, superficial wound infections.

As grade 2 complications were classified, those requiring a more complex supportive treatment than the previous, such as blood transfusions or infection requiring specific antibiotic therapy.

 In grade 3, those complications requiring endoscopic or surgical maneuvers, performed under anesthesia, were classified for resolution. In our study, there was an endoscopic hemostasis for variceal bleeding or secondary suture for dehiscent wound under local anesthesia.

 Grade 4 have included the life-threatening complications requiring supportive care in the intensive care ward - kidney or liver dysfunctions, isolated or associated.

 Death was coded as grade 5. See **Table 2**.

**Table 2:** Complications for Grade 1, 2, 3, 4, 5

Complications		Child A	Child B	Child C
	Hematoma		1	
Grade 1	Seroma	1		
	Superficial wound infection	1	1	
	Erosive gastritis (melena)		1	
Grade 2	Dynamic ileus		1	
	Infection requiring specific antibiotic therapy		2	1
Grade 3	Variceal bleeding		1	
	Wound dehiscence		1	1
Grade 4	Kidney dysfunction		1	
	Liver dysfunction		1	1
Death (grade 5)			1	4

There were 5 deaths in the group of studied patients, representing 22,8%.

The statistical data were obtained by using simple analysis of variance with an α value of 0,05. P-value was 0,0004.

## Results 

The 22 patients of the study group were followed and quantified for postoperative complications, which occurred in a period of 30 days. There were 5 deaths, accounting for 22.2%. See **Table 2**. The complications recorded in patients of Child A class were mild, as grade 1, according to Clavien classification. A seroma and a wound infection have been noted, both requiring only a local treatment.

All the patients in Child B class had complications at all levels. Two of them (18.2%) were grade 1 - one hematoma and one wound infection.

Four (36,4%) grade 2 complications were recorded in this group. One patient presented postoperative melena caused by erosive gastritis, requiring blood transfusion associated with gastric antisecretory and hemostatic treatment.

 One patient presented a dynamic ileus, requiring the installation of a nasogastric tube and correcting electrolyte imbalance. Bowel movements resumed in the 5th postoperative day.

 Two patients had extensive wound infections and required antibiotics therapy.

 Grade 3 complications recorded in Child B stage patients were: one upper GI bleeding with variceal etiology that required endoscopic hemostasis, and one operative wound dehiscence, which was solved by secondary suture practiced under local anesthesia (18.2%).

There were two severe complications (18.2%) in Child B patients group, such as grade 4, represented by acute renal failure - 1 case and hepatic failure - 1 case both requiring resuscitative measures in the intensive care unit.

One death (9%) was registered in this group.

 Out of the 7 patients in Child class C, four died (57.1%) and there were three complications: one severe (14.3%) represented the hepatorenal syndrome, one grade 3 -wound dehiscence resolved by secondary suture and one grade 2 - a severe infection that required antibiotics.

No complications caused by operative gestures applied to saccular contents in strangulation cases were recorded. The two cases that required ileal loop resection with mechanical anastomosis, both in Child B stage, recorded 2 complications both of grade 2 - one dynamic ileus and one wound infection.

There were no hernia recurrences during patients’ follow-up.

 According to Child-Pugh-Turcotte classification, grouping patients in the evolutionary stages of liver cirrhosis, by reference to the recorded level of complications, we compared the postoperative results by using a simple dispersion analysis, for an α value of 0.05.

**Fig. 4:**
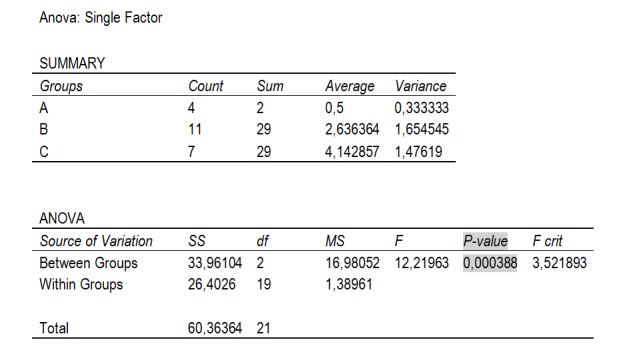
Simple dispersion analysis for patients in different stages of liver cirrhosis

 The results are shown in **Fig. 4**.

## Discussion 

Although considered a small-scale surgery [**10,11**], umbilical hernia repair raises difficulties in therapeutic management in patients with liver cirrhosis.

 The attitude in umbilical hernia in cirrhotic patients is eclectic.

 There are supporters of the resolution under elective surgery even in patients with decompensate disease, resulting in an improvement in their quality of life, provided that the ascites can be effectively controlled [**12**].

Others only support elective surgery in stage Child A; for Child B and C stages the surgical indication remains only in case of complications [**13**].

 On the other side there are those who indicate the hernia repair only in cases of necessity imposed by the occurrence of complications [**14**] because of the high rate of morbidity and mortality associated with the advanced liver disease [**15**].

 In the studied group of patients, surgery was performed in emergency conditions under spinal, 5 cases, or local anesthesia, 17 cases, the procedure being imposed by strangulation, incarceration or ruptured umbilical hernia.

 Our option in the operative procedure was the tissular herniorrhaphy with interrupted sutures by using polypropylene 1.

 No prosthetic materials have been used, in order to avoid the high rate of infection associated with this procedure in this category of patients [**14**].

Eight cases (36.4%) have required surgical gestures on saccular content, requiring 6 partial omentectomies and two segmental resection of the small bowel with mechanical anastomosis. There were no complications related to these interventions. Complications and mortality rate was higher in advanced stages of liver cirrhosis. The 5 deaths belonged to Child B (1 case - 20%) and Child C (4 cases - 80%) stages.

 Postoperative morbidity mainly belonged to Child B and C stages, wound-related complications and general complications such as hepatic decompensation, renal or gastrointestinal bleeding predominating.

## Conclusions 

Although considered a small-scale intervention, liver cirrhosis umbilical hernia repair remains a procedure with a high morbidity and mortality rate if it is performed in emergency conditions due to the occurrence of complications. There is an increased incidence of complications and severity in patients who are in advanced stages of disease, Child B and C.

These assumptions being given and the bad prognosis in advanced liver stages of disease, it is advisable to address the hernia repair in elective conditions in patients who are in the early stages of cirrhosis, compensated liver disease, and avoid complications that occur in emergency conditions.
